# ATP sulfurylase atypical leucine zipper interacts with Cys3 and calcineurin A in the regulation of sulfur amino acid biosynthesis in *Cryptococcus neoformans*

**DOI:** 10.1038/s41598-023-37556-5

**Published:** 2023-07-20

**Authors:** Jeyson Pereira da Silva, Mariana Reis Meneghini, Ronaldo Silva Santos, Verônica Lira Alves, Kevin Felipe da Cruz Martho, Marcelo Afonso Vallim, Renata Castiglioni Pascon

**Affiliations:** Universidade Federal de São Paulo, Campus Diadema, Rua São Nicolau, Diadema, SP 21009913-030 Brazil

**Keywords:** Fungi, Microbial genetics, Pathogens

## Abstract

Fungal pathogens are a major cause of death, especially among immunocompromised patients. Therapies against invasive fungal infections are restricted to a few antifungals; therefore, novel therapies are necessary. Nutritional signaling and regulation are important for pathogen establishment in the host. In *Cryptococcus neoformans*, the causal agent of fungal meningitis, amino acid uptake and biosynthesis are major aspects of nutritional adaptation. Disruptions in these pathways lead to virulence attenuation in an animal model of infection, especially for sulfur uptake and sulfur amino acid biosynthesis. Deletion of Cys3, the main transcription factor that controls these pathways, is the most deleterious gene knockout in vitro and in vivo, making it an important target for further application. Previously, we demonstrated that Cys3 is part of a protein complex, including calcineurin, which is necessary to maintain high Cys3 protein levels during sulfur uptake and sulfur amino acid biosynthesis. In the current study, other aspects of Cys3 regulation are explored. Two lines of evidence suggest that *C. neoformans* Cys3 does not interact with the F-box WD40 protein annotated as Met30, indicating another protein mediates Cys3 ubiquitin degradation. However, we found another level of Cys3 regulation, which involves protein interactions between Cys3 and ATP sulfurylase (*MET*3 gene). We show that an atypical leucine zipper at the N-terminus of ATP sulfurylase is essential for physical interaction with Cys3 and calcineurin. Our data suggests that Cys3 and ATP sulfurylase interact to regulate Cys3 transcriptional activity. This work evidences the complexity involved in the regulation of a transcription factor essential for the sulfur metabolism, which is a biological process important to nutritional adaptation, oxidative stress response, nucleic acid stability, and methylation. This information may be useful in designing novel therapies against fungal infections.

## Introduction

Methionine and cysteine are crucial amino acids for cellular metabolism because they are incorporated into the polypeptide during protein synthesis. In addition to its proteinogenic nature, cysteine is one of the main precursors of glutathione, a molecule essential to counteract oxidative stress promoting antioxidant defense and maintain redox balance^1^. Methionine is the main donor of the methyl group during the methyl cycle that converts methionine into S-adenosylmethionine (SAM). SAM is used in all methylation reactions that occur in the cell, including DNA methylation, indicating a possible role for sulfur amino acid biosynthesis in epigenetics^2–4^. Methionine is important for polyamine synthesis, which is also important for nucleic acid stability^4^.

In yeast, methionine acts as a sentinel metabolite during nutritional stress. Its shortage is a cue to cell entry into autophagy. Methionine also triggers one carbon metabolism and nucleotide synthesis^1^. Its supplementation signals growth, inhibits autophagy, regulates tRNA thiolation, and controls the metabolic state, leading to cellular proliferation. Methionine restriction also correlates to extended lifespan in various model organisms^5^.

In the opportunistic fungal pathogen *C. neoformans,* the sulfur uptake and sulfur amino acid biosynthetic pathway has been studied from the virulence perspective^6–12^. The inability to synthesize sulfur amino acids, either by interruptions in the biosynthetic route or by deregulation of the pathway by deletion of Cys3, which is its main transcription activator, has long been associated with deficiency in virulence factors and attenuated virulence in an animal model of infection^6,7,9^. While *met*3Δ (ATP sulfurylase) and *met*6Δ (methionine synthase) have attenuated virulence, these auxotrophs of sulfur amino acids can be complemented with methionine and cysteine^9^. *C. neoformans cys*3Δ mutant is also avirulent in vivo but can only be complemented with sulfur amino acid supplementation under nitrogen catabolism repression alleviated by proline as a nitrogen source. However, even in this nutritional condition, *cys*3Δ has very poor growth. In fact, *cys*3 deletion was considered lethal in the past, until our group found the necessary conditions to rescue a knockout mutant; therefore, it is considered a conditionally essential gene^6^. The pleiotropic effects of *cys*3Δ mutation make it an appealing drug target; thus, strategies to block its transcriptional activity may be valid.

*C. neoformans* Cys3 is a BZip transcriptional regulator (MetR and Met4 in *Neurospora crassa* and *S. cerevisiae*, respectively) that contains a conserved basic region, a nuclear localization signal, followed by a classical leucine zipper. Cys3 is responsible for the induction of many genes in all branches of the biosynthetic pathway in response to the nutritional condition^6^. In *C. neoformans*, *S. cerevisiae*, *Aspergillus nidulans,* and *N. crassa*, methionine and cysteine supplementation leads to Cys3/MetR/Met4 protein extinction^3,6,13–16^. A regulatory hub in this pathway is represented by an F-box protein (Met30), which was first described in *N. crassa* (Scon1 and 2) as important down regulators of the sulfur metabolism and methionine biosynthesis^3,13^. Since Met30 is part of the ubiquitin-ligase pathway, it is responsible for down-regulating Met4/MetR activity by targeting it for degradation^17–19^. There is a homologue of Met30 (CNAG_05773) in *C. neoformans*; however, its role on Cys3 regulation is unknown.

Recently another Cys3 regulatory mechanism was identified in *C. neoformans*. Our previous work showed that the calcineurin complex physically interacts with Cys3 and is required to maintain high levels and its subcellular localization in the nucleus under inorganic sulfur nutritional condition. Deletion of catalytic and regulatory calcineurin subunits leads to loss of Gfp-Cys3 protein and abnormal subcellular localization. Degradation of Gfp-Cys3 protein is followed by down regulation of *SUL*1 sulfate permease, one of its main transcriptional targets^6^.

The protein levels of Cys3 transcription factor are also influenced by other metabolic cues, such as lipid metabolism. The inability of cells to produce glycerol due to glycerol phosphate phosphatase gene deletion (Gpp2) leads to abnormal accumulation of Cys3 transcription factor and a persistent subcellular localization to the nucleus, even in the presence of organic sulfur source^6,20^. This could also be linked to NADPH reductive capacity of the cell, since methionine biosynthesis is considered one of the largest sinks for NADPH consumption^1,21^, which may be deregulated in *gpp*2Δ mutant; however, that hypothesis remains to be tested.

Our previous work showed that Cys3 is part of a large protein complex. Some of the interactions found have been elucidated and shown to be relevant for the fungal metabolism^6,9^, others remain to be confirmed. Previously, we reported an interaction between Cys3 transcription factor and ATP sulfurylase found by immunoprecipitation followed by mass spectrometry^6^. ATP sulfurylase, encoded by *MET*3 gene, conducts the first committed step of sulfur assimilation after sulfate uptake by permeases Sul1 and Sul2, yielding adenosine phosphosulfate (APS).

The present paper explores Cys3 interactions with ATP sulfurylase (Met3), which may have regulatory roles on sulfur uptake and sulfur amino acid biosynthesis. Even though our previous IP-LC/MS experiments did not show Cys3 and Met30 in a protein complex, we tested the hypothesis that Cys3 would physically interact with Met30 for pathway down regulation. However, these proteins did not interact in *S. cerevisiae* two-hybrid assay. In this work, we show that Cys3 interacts with ATP sulfurylase through an atypical leucine zipper to maintain Cys3 protein stability. The lack of *MET*3 gene leads to Gfp-Cys3 abnormal processing. Furthermore, we detected an interaction by two-hybrid assay between calcineurin A and ATP sulfurylase, which seems to be essential for ATP sulfurylase accumulation and stability, similar to previously reported dependency of Cys3 on calcineurin A and B^6^.

The data collected in this work significantly contribute to the idea that Cys3 is the target of an intricate regulatory mechanism with multiple layers of transcriptional and post-transcriptional controls, aiming to maintain sulfur uptake and sulfur amino acid biosynthesis under tight control.

This work not only presents a novel mechanism of Cys3 regulation, but also highlights a possible strategy for pathogen control, since the Cys3 is the key regulator of the sulfur amino acid biosynthetic route and is essential for growth, virulence, and pathogenesis of *C. neoformans*.

## Results

### ATP sulfurylase interacts with Cys3 and Cna1 in two-hybrid assay

We identified several Cys3 protein partners by IP-LC/MS; however, among these proteins we were unable to see an equivalent to Met30^6^. Despite this, *C. neoformans* has a *MET*30 gene that encodes a homologue of the F-Box protein component of SCF^Met30^ annotated in the genome (CNAG_05773) (Supplementary Fig. S1). We used a yeast two-hybrid assay to test the interaction between Met30 as bait (pRCP109) and Cys3 as prey (pRCP099). The results showed no interaction between Met30 and Cys3 (Supplementary Fig. S1).

Our previous work found proteins associated to Gfp-Cys3, such as calcineurin A, calcineurin B, Gpp2 phosphatase, and ATP sulfurylase; therefore, a complex regulation for this important transcription factor was discovered. Besides physical interaction, the calcineurin complex and Gpp2 affect the transcriptional ability of Cys3, since its target genes were found deregulated in *cna*1Δ, *cnb*1Δ, and *gpp*2Δ strains^6^. Moreover, ATP sulfurylase encoded by *MET*3 gene was observed in a protein complex with Cys3 by IP-LC/MS (immune precipitation followed by mass spectrometry)^6^. Although this data has not been confirmed by other means, it may also represent a regulatory mechanism for Cys3 activity as a transcription factor.

**Figure 1:**
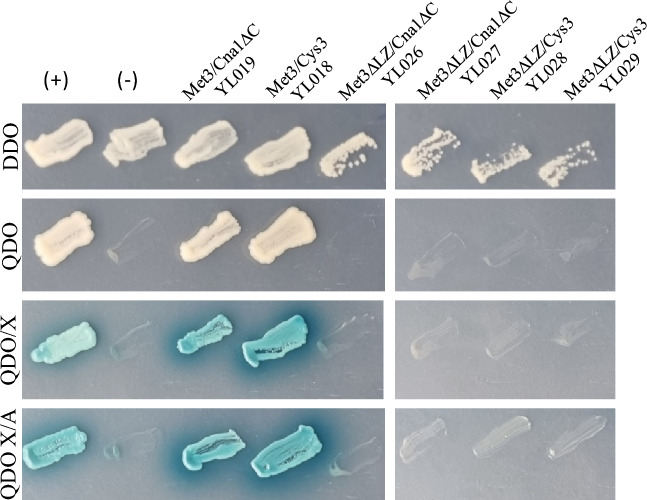
Physical interactions of ATP sulfurylase with Cys3 and Cna1 by two-hybrid analysis. Images show representative colonies of *S. cerevisiae* expressing pairs of bait and prey fusion proteins, which are described at the top. + represents the positive and—the negative control provided by Match Maker kit (Clontech). YL019 is the strain code for *S. cerevisiae* Y2HGold carrying the plasmids that encodes the ATP sulfurylase (Met3) as bait and the catalytic subunit of calcineurin A deleted for the auto inhibition domain Cna1ΔC as prey; YL018: interaction of ATP sulfurylase as bait with Cys3 transcription factor as prey; YL026 and YL027: ATP sulfurylase deleted for the atypical leucine zipper (Met3ΔLZ) as bait and the catalytic subunit of calcineurin A deleted for the auto inhibition domain (Cna1ΔC) as prey; YL028 and YL029: ATP sulfurylase deleted for the leucine zipper (Met3ΔLZ) as bait and the Cys3 transcription factor as prey. Each line represents a different plate composition that test for different reporter genes; DDO = double drop out (minus triptophane and leucine), where no reporter gene is activated and tested; QDO = quadruple drop out (minus tryptophan, leucine, histidine and adenine), where two reporters are tested and QDO/X/A = quadruple drop out added with aurobasidin; and X-α-Gal, where all four reporters are tested. Plates were grown for 96 h at 30 °C, and photographed after this period.

To collect information about the regulation of Cys3, we analyzed the interactions of ATP sulfurylase with the other members of the complex. Two hybrid assays were conducted to confirm the interactions shown by IP-LC/MS. The first four lines in Table 1 summarizes the experiments that were conducted to test the interactions between ATP sulfurylase and Cys3, Gpp2, Cnb1, and Cna1ΔC. The C-terminus region of Cna1 has a calcium-binding domain that changes its conformation during calcium binding. Deletion of this domain allows the binding of Cna1 to its partners in the absence of Ca^2+^/calmodulin; therefore, the construct used in this work has the exact same C-terminus deletion described in the literature^22^, which is identified in this work as Cna1ΔC. Self-activation and toxicity of the pRCP106 (*DB*::*MET*3) were also tested to guarantee the accuracy of the experiment. Table 1 shows the negative and positive physical interactions observed. Interactions between ATP sulfurylase and Cys3 as well as ATP sulfurylase and Cna1ΔC were found. Gene reporter activation can be observed in Fig. 1, where ATP sulfurylase and Cys3 as well as ATP sulfurylase and Cna1ΔC grew on QDO, QDO/X, and QDO/X/A, which suggests these pairs of proteins undergo physical interaction. These results are an independent confirmation of the data obtained by IP-LC/MS.

**Table 1:** Interactions tested in *S. cerevisiae* two-hybrid assay.

Interactions	Interacting proteins	pGADT7/pGBKT7	Self-activation	Interaction
1	Cys3 + Met3	pRCP099/pRCP106 (YL018)	–	Yes
2	Cna1-ΔC + Met3	pRCP094/pRCP106 (YL019)	–	Yes
3	Cnb1 + Met3	pRCP096/pRCP106	–	No
4	Gpp2 + Met3	pRCP088/pRCP106	–	No
5	Cys3 + Met3ΔLZ	pRCP099/pRCP128 (YL028)	–	No
6	Cys3 + Met3ΔLZ	pRCP099/pRCP132 (YL029)	–	No
7	Cna1-ΔC + Met3ΔLZ	pRCP094/pRCP128 (YL026)	–	No
8	Cna1-ΔC + Met3ΔLZ	pRCP094/pRCP132 (YL027)	–	No
9	Positive control ( +)	pGADT7-T/pGBKT-53	–	Yes
10	Negative control (–)	pGADT7-T/pGBKT7-Lam	–	No

### *C. neoformans* ATP sulfurylase has an atypical leucine zipper and putative calcineurin binding site

Since ATP sulfurylase interacts with Cna1 and Cys3, we searched for possible protein domains that could be used as anchors for protein–protein interactions and provide an explanation for this phenomenon.

**Figure 2:**
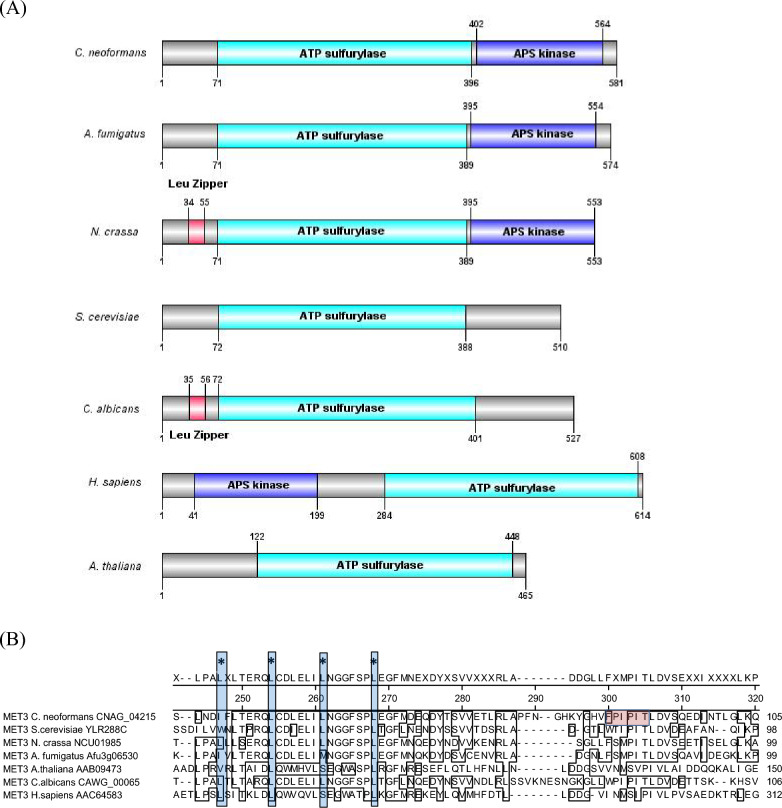
Domains and motifs of ATP sulfurylase in various organisms. (**A**) Schematic representation of the domains present in ATP sulfurylase in *C. neoformans* (CNAG_04215), *A. fumigatus* (Afu3g06530), *N. crassa* (NCU01985), *S. cerevisiae* (YJR010W), *C. albicans* (CAWG_00065), *H. sapiens* (AAC64583), and *A. thaliana* (AAB09473). The numbers indicate the position of the domains. ATP sulfurylase domain is in green, APS kinase in blue, and leucine zipper in red. Image was generated by DOG 1.0: Illustrator of Protein Domain Structures software^41^. (**B**) Alignment of the amino acid sequence of the putative leucine zipper found at the N-terminus of the ATP sulfurylase in various organisms; the four conserved leucine/isoleucine separated by any of the six amino acids are boxed in blue and indicated by asterisk. The putative PxIxIT motif in *C. neoformans* is highlighted in pink. The alignment was generated by MegaAlign Software Lasergene (DNA Star).

*C. neoformans* ATP sulfurylase was first described in the literature by Yang and collaborators^12^. This enzyme is encoded by *MET*3 gene, which is located at chromosome 9. *MET*3 regulation is under the control of Cys3, as shown by our previous report^6^. *C. neoformans* ATP sulfurylase has two main domains: the catalytic domain is predicted to span from amino acids 71 to 396 (Fig. 2A). This region is responsible for the ATP sulfurylation, which is the first intracellular step in this pathway. From amino acid 402 to 581, there is an APS kinase-like domain. However, *C. neoformans* genome encodes an APS kinase (*MET*14 CNAG_02202), which generates the 3′-phospho-5′adenylylsulfate (PAPS). The latter is thought to be the functional APS kinase rather than the one encoded by *MET*3. The ATP sulfurylase of *P. chrysogenum* also has the APS kinase that has no phosphorylation activity^23^.

In the amino acid sequence alignment among several fungi, plants, and humans, the ATP sulfurylase shows that *C. neoformans*, *Aspergillus fumigatus,* and *Neurospora crassa* share the same domain arrangement (Fig. 2A). *S. cerevisiae*, *C. albicans,* and *Arabidopsis thaliana* lack the APS kinase domain, and *Homo sapiens* has both domains, but in opposite orientation as *C. neoformans* (Fig. 2A).

A distinguished feature of ATP sulfurylase is the presence of *bona fide* leucine zipper at the N-terminus, with 4 leucines separated by any 6 amino acids in a row (4x[LxxxxxxL]) in ATP sulfurylase of *C. albicans* and *N. crassa* (Fig. 2B, blue shadow). The alignment also presents this feature in *C. neoformans* and *S. cerevisiae;* however, the first leucine of the zipper is substituted by an isoleucine and a valine, respectively. The leucine zipper is mostly conserved in all other organisms aligned in Fig. 2B with substitutions at the third leucine (*H. sapiens*) and at the first and third leucines for *A. fumigatus* and *A. thaliana* (Fig. 2B).

Following the leucine zipper, there is a putative PxIxIT motif (Fig. 2B red shadow), which has been associated with a calcineurin binding site to the substrates^24^. The best fit for a putative PxIxIT found in *C. neoformans* ATP sulfurylase is ^85^FPIPIT^90^, where the only substitution is the first amino acid of the motif, since a proline is substituted by a phenylalanine. Previously, a transcription factor (Crz1) was found to be positively regulated by calcineurin to promote a cellular response to high temperature growth; however, a PxIxIT motif was never found^25^, indicating that, in this organism, the motif may deviate from the consensus. Other cases of functional atypical PxIxIT domains can be found in the literature^26,27^.

Since Cys3 protein has a classic leucine zipper which serves the purpose of homodimarization to generate a functional transcription factor, we hypothesize that the putative leucine zipper encountered at the N-terminus of ATP sulfurylase may be the domain by which Cys3 and ATP sulfurylase interact with each other, creating a heterodimer.

### An atypical leucine zipper is essential for ATP sulfurylase and Cys3 interaction in a two-hybrid assay

To test the hypothesis that the two proteins interact by their leucine zippers, we deleted the atypical leucine zipper from ATP sulfurylase and fused the *MET*3ΔLZ allele to the Gal4 DNA binding domain in pGBKT7 plasmid. This deletion involved 66 nucleotides encoding 22 amino acids of the zipper (from Ile^34^ to Leu^55^ Supplementary Fig. S2) and generated two independent plasmids pRCP128 and pRCP132 (pGBKT7 containing BD::*MET*3ΔLZ gene fusion). The deletion was confirmed by DNA sequence of the plasmids (supplementary Fig. S3). Then, pRCP128 and pRCP132 (pGBKT7 containing *BD*::*MET*3ΔLZ gene fusion) were separately introduced in Y2HGold along with pRCP099 (pGADT7 *AD*::CYS3), and two *S. cerevisiae* clones (YL028 and YL029) were tested for reporter activation. Table 1 and the right side of Fig. 1 shows that the interaction was lost with the deletion of the leucine zipper, as compared to YL018 in which the *MET*3 sequence fused to DB domain is a wild-type allele that retains the atypical leucine zipper. This result confirms that ATP sulfurylase not only interacts with Cys3, but it strongly suggests that the interaction occurs through the atypical leucine zipper at the N-terminus of the ATP sulfurylase.

Since a two-hybrid interaction between Met3 and Cna1ΔC was found, we also tested if the interaction between Met3ΔLZ and Cna1ΔC would be affected by the deletion of the atypical leucine zipper. As shown in Table 1 and Fig. 1, strains YL026 and YL027 failed to induce the reporter genes, indicating that the interaction was lost due to the atypical leucine zipper deletion. However, in strain YL019, all 4 reporter genes (QDO/X/A) were activated, and in this case, ATP sulfurylase carried the atypical leucine zipper.

These results showed that these proteins not only physically interact with each other, but ATP sulfurylase and Cys3 also undergo protein–protein interactions through the leucine zipper domain of the ATP sulfurylase, even though the zipper encountered at ATP sulfurylase is not a *bona fide*.

### Gfp-Met3 is a cytoplasm protein

Our hypothesis was that Cys3 and Met3 interact through the atypical leucine zipper to regulate Cys3 activity, and we predicted that ATP sulfurylase is a cytoplasm protein. However, there is no data on the cellular localization of ATP sulfurylase in *C. neoformans* to support this hypothesis.

In the literature, multiple cellular localizations of ATP sulfurylase have been proposed for different organisms^28^. In *A. thaliana* and other photosynthetic eukaryotic organisms, such as green algae, it has been found in plastids and in the cytosol^29,30^. On the other hand, human ATP sulfurylase may occur in the nucleus and the cytoplasm of eukaryotic cells^31^.

**Figure 3:**
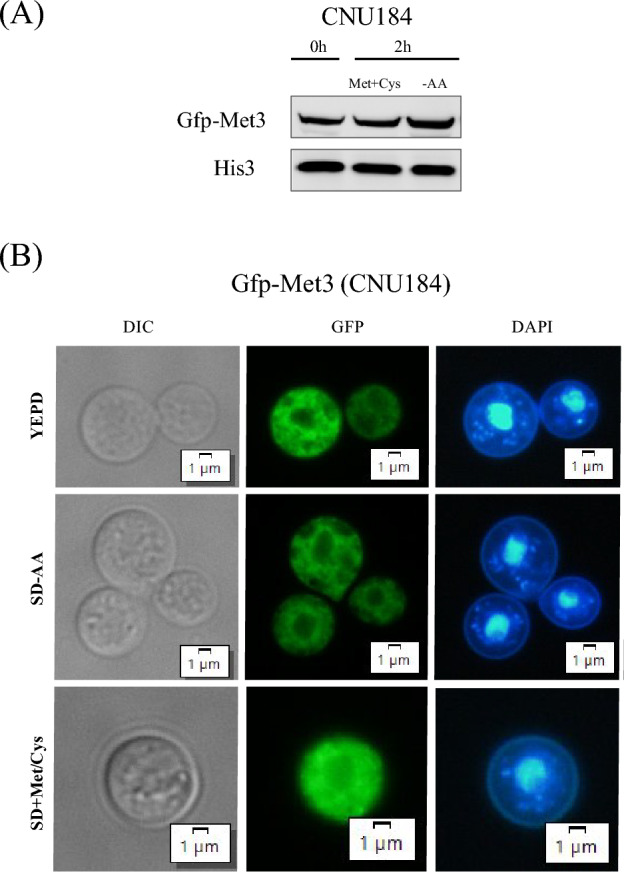
Western blot and fluorescent microscopy of Gfp-Met3. (**A**) Western blot of total proteins extracted from strain CNU184 cultivated for 2 h in SD and SD-N + Cys/Met. Mouse Anti-GFP primary antibody was used at 1:7000 dilutions and secondary anti-mouse horseradish peroxidase-linked antibody was applied at 1:2000 dilutions. Loading control was done by detection of Histone H3 protein with rabbit anti-His3 antibody (1:2000) as primary and anti-rabbit secondary antibody horseradish peroxidase linked (1:2000). (**B**) Bright field (BF) and fluorescent microscopy of Gfp-Met3 (GFP) and nuclear staining (DAPI) in two independent strains containing the *GFP-MET*3 allele integrated at the genome (CNU184). Western blot and fluorescent microscopy images are representative of CNU184 and CNU185 individual mutants.

To describe the cellular localization of ATP sulfurylase in *C. neoformans*, the GFP nucleotide sequence was fused to the coding and terminator sequences of the *MET*3 gene, generating plasmid pRCP120. This construct was introduced in the wild-type strain H99, generating two independent strains (CNU184 and CNU185) that presented a specific 91 KDa band, which is the expected molecular weight for the fusion protein Gfp-Met3 in western blot (Fig. 3A).

Supplementation of the medium with methionine and cysteine did not change Gfp-Met3 band pattern in the western blot (Fig. 3A), suggesting that ATP sulfurylase is not subjected to sulfur amino acids regulation, as it is the case for Cys3^6^.

The same strains were observed under fluorescent microscopy (Fig. 3B). In all conditions (YEPD, SD supplemented with ammonium sulfate minus and plus sulfur amino acids), the Gfp-Met3 protein was found to be evenly distributed throughout the cytoplasm and excluded from the nucleus (Fig. 3B). This result confirms that in *C. neoformans* ATP sulfurylase is a cytoplasm protein that interacts with Cys3 by the leucine zipper. This interaction might act as a regulatory mechanism that fine tunes Cys3 activity during the sulfur assimilation and sulfur amino acid biosynthesis.

### Deletion of *MET*3 gene changes the Gfp-Cys3 pattern in western blot and fluorescent microscopy

We showed by independent experiments that ATP sulfurylase and Cys3 undergo protein interaction. The *S. cerevisiae* two-hybrid assay demonstrated that this interaction is mediated by the atypical leucine zipper present in ATP sulfurylase, a cytoplasm protein. We predict that this interaction occurs in *C. neoformans* as a way to regulate Cys3 levels.

*C. neoformans* CNU080 strain carries a *GFP::CYS*3 allele integrated in its genome and expresses a fusion protein (Gfp-Cys3) that accumulates largely in the nucleus in the presence of inorganic sulfur source, especially in complex medium (YEPD), leading to increased expression of several genes of the sulfur uptake pathway and sulfur amino acid biosynthesis^6^. The supplementation of the medium with methionine and cysteine leads to a decrease in Gfp-Cys3 band in western blot, and in a large percentage of cells, the Gfp-Cys3 protein exits the nucleus^6^. Therefore, we created a *met*3Δ mutant in the CNU080 strain (Gfp-Cys3) by CRISPR-Cas9 technology to test if the lack of ATP sulfurylase changes the stability and localization of the Gfp-Cys3 fusion protein.

**Figure 4:**
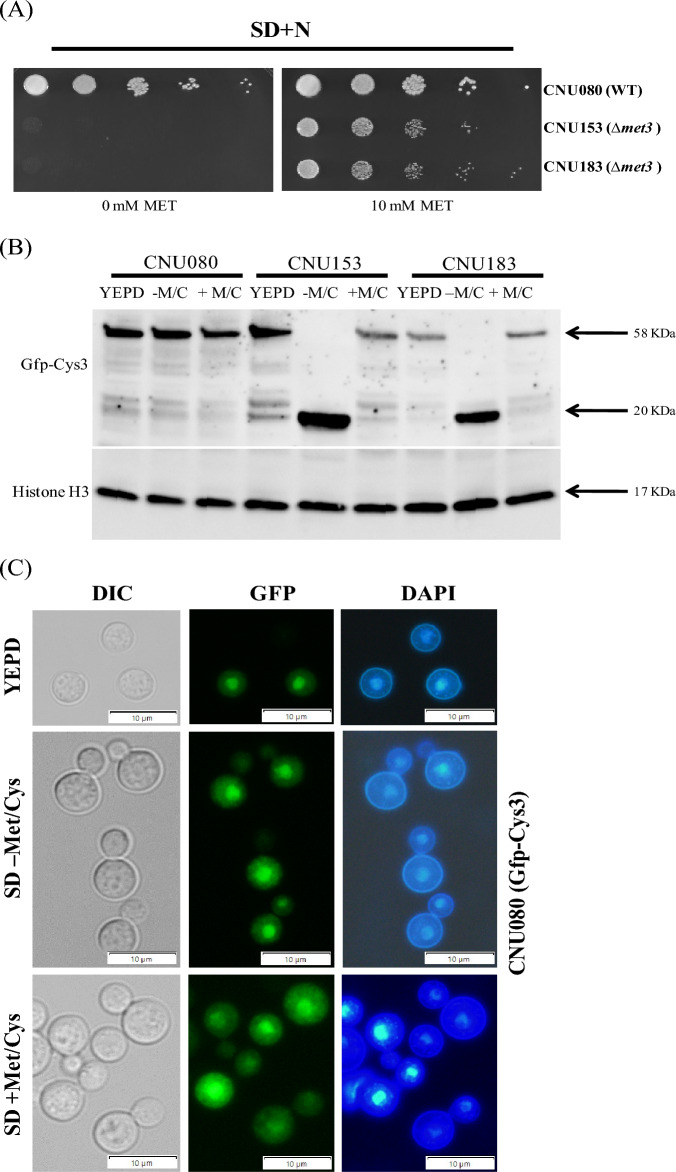

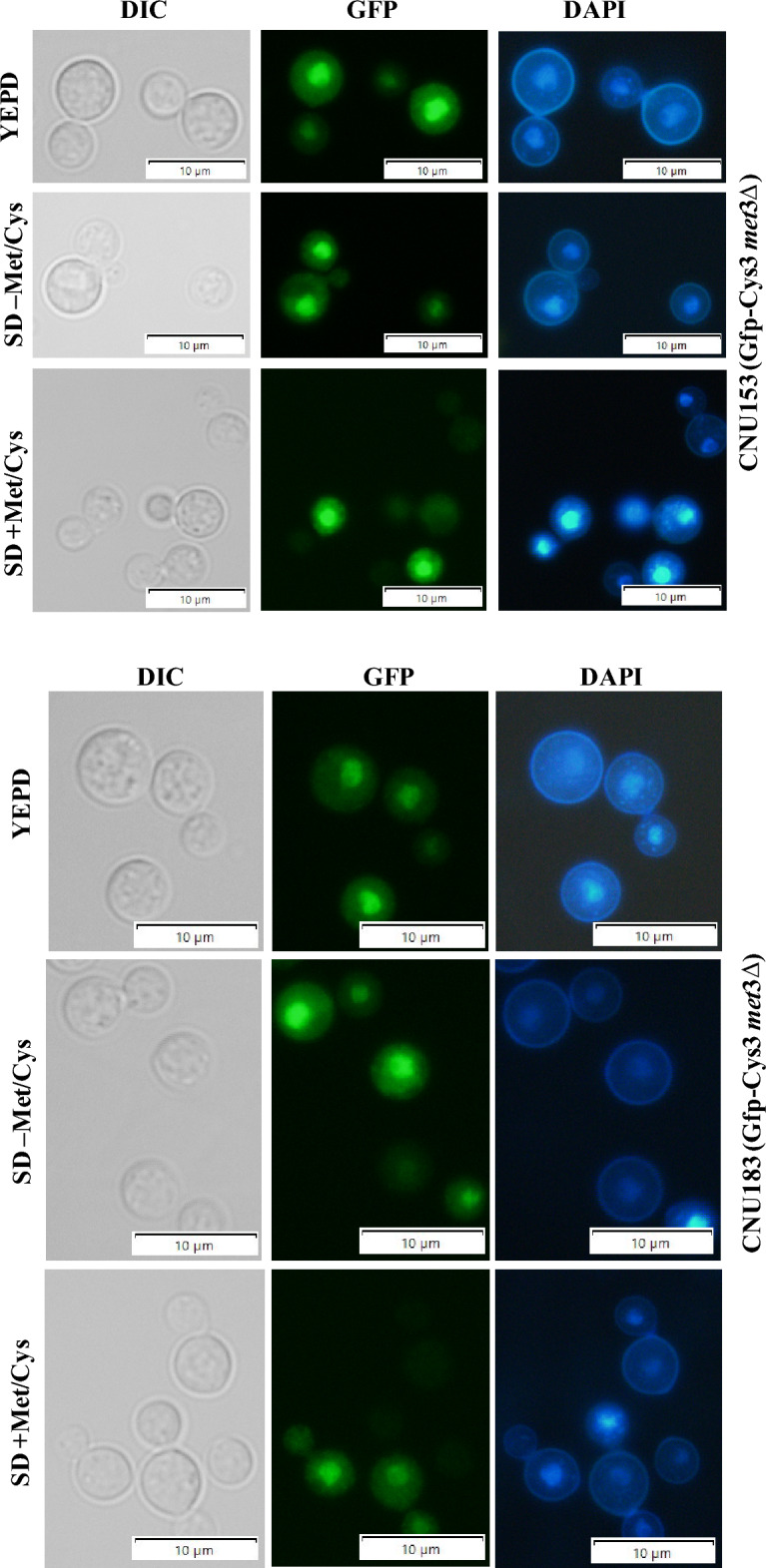

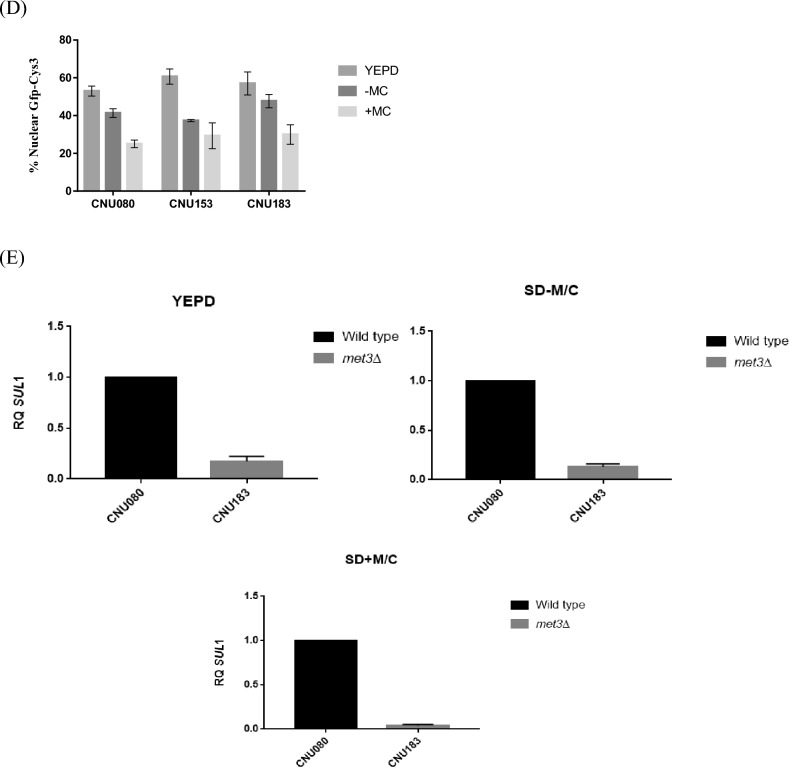
Cys3 protein localization and expression under *met*3Δ deletion background. (**A**) CRISPR-Cas9 was used to generate a *met*3Δ deletion in the host strain (CNU080), which expresses Gfp-Cys3 fusion protein. The mutant strains CNU153 and CNU183 (Gfp-Cys3 *met*3Δ::*Nat*^R^) are sulfur amino acid auxotrophic as shown by their lack of growth in SD compared to the wild type (CNU080); in SD + Met/Cys, all 3 strains grew. (**B**) The three strains were cultivated for 2 h in three conditions (YEPD, SD – M/C, and SD + Met/Cys) at 30 °C before total protein extracts. SDS-PAGE and western blots were made. Mouse Anti-GFP primary antibody was used at 1:7000 dilutions and secondary anti-mouse horseradish peroxidase-linked antibody was applied at 1:2000 dilutions. Loading control was done by detection of histone H3 protein with rabbit anti-His3 antibody (1:2000) as primary and anti-rabbit secondary antibody horseradish peroxidase linked (1:2000); (**C**) Images of florescent microscopy of the strains CNU080 (wild type), CNU153 (Gfp-Cys3 *met*3Δ::*Nat*^R^), and CNU183 (Gfp-Cys3 *met*3Δ::*Nat*^R^) were conducted in bright field (BF), GFP fluorescence detecting Gfp-Cys3 (GFP), and nuclear staining (DAPI); strains were cultures in YEPD, SD supplemented with ammonium sulfate (SD–Met/Cys), and SD supplemented with ammonium sulfate and 20 mM sulfur amino acids (SD + Met/Cys); (**D**) percentage of nuclear Gfp-Cys3 co-localized with DAPI staining in CNU080, CNU153, and CNU183 in three different nutritional conditions (YEPD, SD supplemented with ammonium sulfate plus and minus methionine and cysteine); n > 100/strain/nutritional condition; experiments were done in biological replicates; (**E**) differences in expression patterns of *SUL*1 in wild-type (CNU080) and mutant strain *met*3Δ (CNU183) cultivated in YEPD, SD – M/C, and SD + M/C are statistically significant (*p* = 0.0007, *p* = 0.0150, and *p* < 0.0001, respectively); RQ means relative quantification.

We were able to rescue several transformants that were auxotrophs for sulfur amino acids, and two of them (CNU153 and CNU183) were selected for further analysis (Fig. 4A). The sulfur amino acid growth deficiency could be completely supplemented either with methionine or cysteine; however, the *cys*3Δ mutant could only barely be satisfied by cysteine supplementation and has decreased growth rate.

Protein extracts from wild-type Gfp-Cys3 (CNU080) and mutants Gfp-Cys3, *met*3Δ::*Nat*^R^ (CNU153 and CNU183) were obtained in 3 different conditions after two hours in: YEPD, in SD + (NH_4_)_2_SO_4_ (SD-M/C), and SD + (NH_4_)_2_SO_4_ + Methionine and Cysteine (SD + M/C). As shown in Fig. 4B, when growth was carried out in YEPD, all 3 strains presented the typical 58 KDa band which is consistent with the molecular weight of Gfp-Cys3 fusion protein. Under growth in SD supplemented with methionine and cysteine, the band intensity decreases compared to YEPD in all 3 strains, as expected according to our previous data^6^; however, this effect is more intense in both mutants (Fig. 4B). In SD supplemented with only ammonium sulfate, the 58 KDa Gfp-Cys3 band was extinguished; however, an approximately 27 KDa band was generated, which is consistent to the molecular weight of Gfp. This band pattern is very consistent in both independent mutants (CNU153 and CNU183 Fig. 4B). This result suggests that ATP sulfurylase is necessary for Cys3 stability. The lack of the partner protein apparently leads to Cys3 processing in the presence of inorganic sulfur (ammonium sulfate). In the presence of sulfur amino acid, the 58 KDa band is less intense, but it is not fully processed and likely undergoes a different kind of degradation that does not produce the 27 KDa band.

Fluorescent microscopy analysis of these strains found no statistically significant difference between wild-type and mutant strains in the percentage of nuclear Gfp-Cys3 (Fig. 4C,D). As expected for the wild type, the highest percentage of nuclear Gfp-Cys3 is in YEPD and SD–M/C, and this percentage is reduced in the presence of organic sulfur (SD + M/C, Fig. 4D). This pattern of nuclear Gfp-Cys3 is not significantly changed in the mutants, indicating that the altered band pattern for the mutants in western blot (Fig. 4B) does not reflect in subcellular localization (Fig. 4C,D). We also analyzed the ratio of Gfp fluorescence intensity nucleus/cytoplasm and found no statistical difference (Supplementary Fig. S4).

Our previous results demonstrated that reduced levels of Cys3 due to the presence of organic sulfur or deletions of calcineurin A and B trigger transcriptional repression of its targets, such as *SUL*1^6^. Therefore, we asked if deletion of *MET*3 gene would change the transcriptional ability of Cys3 due to its abnormal processing, which is shown on the western blot (Fig. 4B). Figure 4E illustrates reduced levels of *SUL*1 in *met*3Δ strain (CNU183) compared to wild type (CNU080) in all the three conditions tested (YEPD, SD–M/C and SD + M/C).

Taken together, these results demonstrate that ATP sulfurylase is required for proper Cys3 protein processing and that abnormal Cys3 cleavage has consequences in its transcriptional function, rendering Cys3 unable to properly active the transcription of *SUL*1, a main target.

### Deletion of calcineurin a leads to decreased levels of Gfp-Met3 in western blot and fluorescent microscopy

Since we constructed a *C. neoformans* strain that expresses the Gfp-Met3 fusion protein and to show that the interaction of Met3 with Cna1ΔC is not only a physical interaction mediated by Cys3 that places these proteins in a complex, but rather the interaction has consequences on the ATP sulfurylase, we introduced the Gfp-Met3 construct (pRCP120 plasmid) into the *cna*1Δ mutant (CNU166), a strain deleted for the catalytic subunit of calcineurin. We expected to see differences in the band pattern between wild type and mutant, if calcineurin leads to any modifications or if it is part of a mechanism that modulates ATP sulfurylase.

**Figure 5:**
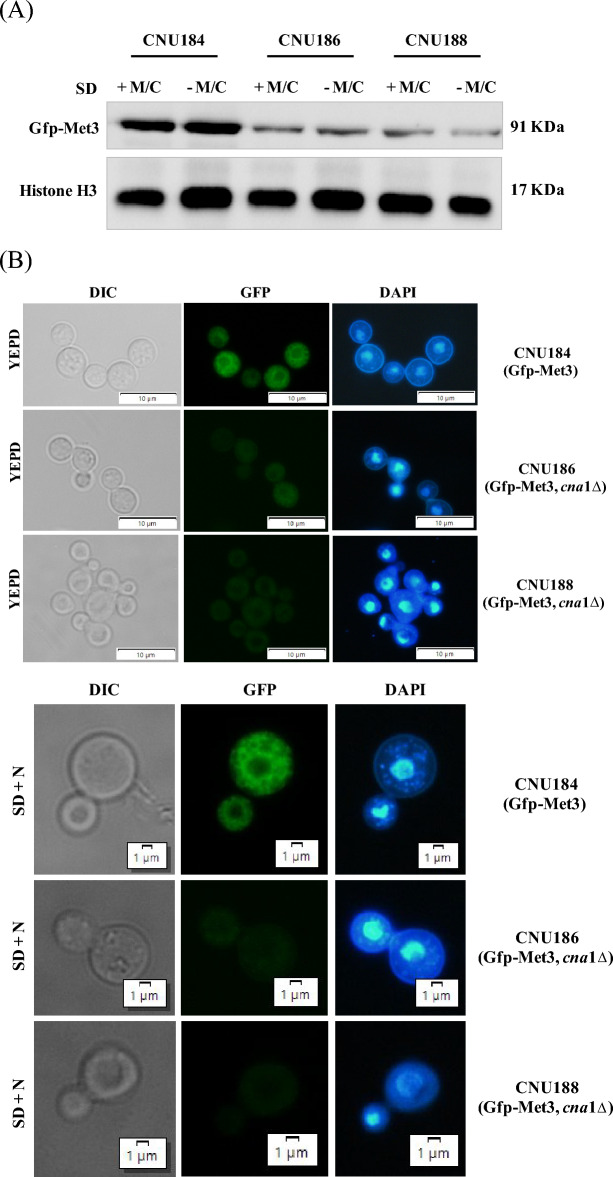

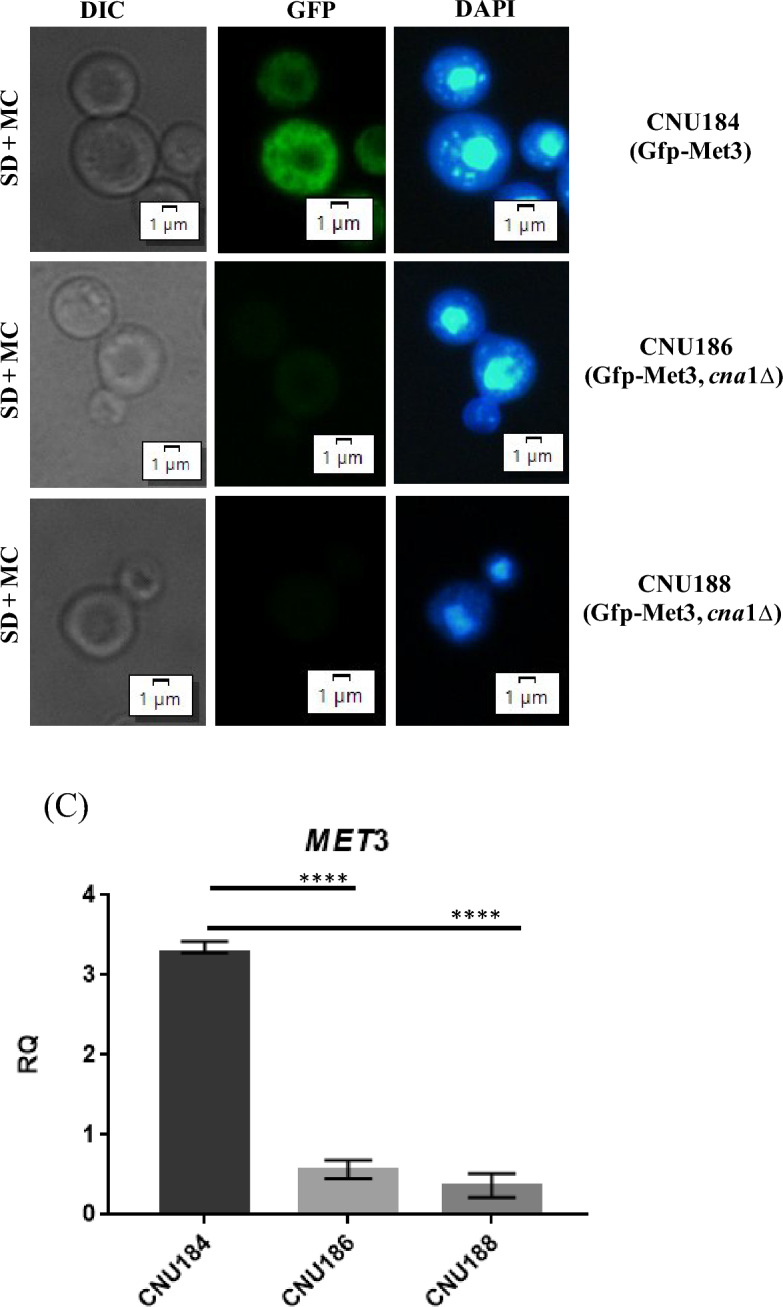
Gfp-Met3 localization and expression in a *cna*1Δ mutant strain. (**A**) Western blot showing the expression pattern of the strain containing the *GFP-MET*3 allele (CNU184) and two independent mutants *cna*1Δ expressing the *GFP-MET*3 allele (CNU186 and CNU188); strains CNU184, CNU 186, and CNU188 were cultivated for 2 h in SD and SD-N + Cys/Met and total proteins were extracted. Mouse Anti-GFP primary antibody was used at 1:7000 dilutions and secondary anti-mouse horseradish peroxidase-linked antibody was applied at 1:2000 dilutions. Loading control was done by detection of histone H3 protein with rabbit anti-His3 antibody (1:2000) as primary and anti-rabbit secondary antibody horseradish peroxidase linked (1:2000); (**B**) Bright field (BF) and fluorescent microscopy of Gfp-Met3 (GFP) and nuclear staining (DAPI) of the CNU184 strain, expressing Gfp-Met3 and two independent strains (CNU186 and CNU188) containing the *GFP-MET*3 allele and deletion for the catalytic subunit of the calcineurin (*cna*1Δ). (**C**) Real time quantitative PCR of *MET*3 gene expression between wild type (CNU184) and *cna*1Δ mutants (CNU186 and CNU188). RQ = relative quantification. Asterisks denote different levels of statistical significance (*p* value < 0.0001).

As shown in the western blots (Fig. 5A), the 91 KDa bands corresponding to Gfp-Met3 in two independent *cna*1Δ mutants (CNU186 and CNU188) are much less intense than in the wild type (CNU184). This result suggests that lack of calcineurin A renders ATP sulfurylase an unstable protein, which is more likely to degrade than in the wild-type background. Since the calcineurin complex has a phosphatase activity, dephosphorylation might be a mechanism to keep ATP sulfurylase out of the reach of protein degradation machinery. A similar observation was reported by de Melo and collaborators (2019) who detected an early extinction of Cys3 protein in the background of *cna*1Δ and *cnb*1Δ strains even in the presence of inorganic sulfate^6^.

Florescence microcopy of the same strains (wild type—CNU184 and *cna*1Δ—CNU186 and CNU188) shows the reduction in the 91 KDa band is reflected in cellular fluorescence of the mutants. The wild-type cells are brighter than mutant cells (Fig. 5B) in all conditions tested (YEPD, SD –M/C, and SD + M/C). However, there is no change in subcellular localization of the Gfp-Met3 fusion protein, which appeared dispersed in the cytoplasm of the wild-type and the mutant strains.

We also performed qPCR to check if *MET*3 gene had altered expression, since we expected from our previous work that deletion of the calcineurin complex would lead to lower *SUL*1 levels. As presented in Fig. 5C, *MET*3, one of the main targets of Cys3, is down regulated in the mutants (CNU186 and CNU188) compared to wild-type strain (CNU184), as expected.

## Discussion

We study multiple regulatory layers of sulfur uptake and sulfur amino acid biosynthesis aiming to understand this mechanism, which may have therapeutic value as a drug target, since impairments in these pathways are deleterious for pathogens, such as *C. neoformans* and *A. fumigatus*^9,12,32,33^. In this paper, we excluded the regulatory role of Met30 encoded by CNAG_05773 in this process, since it did not interact with Cys3, as reported in other organisms, including *S. cerevisiae* and *N. crassa*^13,14,18,19,34–36^. However, at least five other F-Box proteins are encoded by *C. neoformans* genome; therefore, another gene could encode Met30 to target Cys3 to proteosome degradation.

This study showed that *C. neoformans* has different ways to regulate sulfur uptake and sulfur amino acid biosynthesis. Based on previous and present data we can highlight three important points about the metabolic regulation of this pathway in *C. neoformans.* First, sulfur uptake and sulfur amino acid biosynthesis are complex metabolic processes regulated by the sulfur source available in the environment^6^. Cys3 is the main target of this nutritional regulation at both the transcriptional level and the protein level, and it induces transcription of many target genes on the pathway under inorganic sulfur. On the one hand, Cys3 protein is extinguished in the presence of organic sulfur; on the other hand, ATP sulfurylase does not seem to be regulated by the sulfur source at the protein level (Fig. 3). However, it is known that *MET*3 is regulated at the transcriptional level by Cys3 in *C. neoformans*. Second, calcineurin complex is involved in the regulation of Cys3 and ATP sulfurylase at the protein level, likely through its dephosphorylation activity. Although Cnb1 interacts with Cys3, the deletion of both subunits genes (*CNA*1 and *CNB*1) leads to Gfp-Cys3 abnormal degradation and also causes down regulation of one of Cys3 transcriptional targets: the sulfate permease Sul1^6^. In this work, we observed that Cna1 also interacts with ATP sulfurylase (Fig. 1), suggesting that calcineurin complex indeed regulates sulfur uptake in at least two important steps of the pathway: by interfering with Cys3, and at the enzyme level, by interacting the ATP sulfurylase. This illustrates how the calcineurin activity is important for this pathway, since it is regulated by the complex at Cys3 and ATP sulfurylase levels, which are two players with very different molecular functions. This strengthens the idea that calcineurin is required to maintain a tight regulation over the pathway by stabilizing Cys3 and ATP sulfurylase (Fig. 5). Third, we unraveled, for the first time, another regulatory layer. In this case, a physical interaction of Cys3 with ATP sulfurylase through the atypical leucine zipper present in the ATP sulfurylase (Figs. 1 and 2) is required to regulate Cys3, to avoid its processing and to modulate its transcriptional activity (Fig. 4).

Our data also suggests that calcineurin and ATP sulfurylase regulations promote different outcomes of Cys3. In the first case, the protein is extinguished, and in the second case, Cys3 seems to be processed. This is suggestive of two different regulatory processes over the same transcription factor, one triggered by calcineurin complex and the other involving ATP sulfurylase.

Taken together, these results are interesting as we look for pharmacological ways to interrupt important metabolic processes that can lead to fungicidal activity. The sulfur amino acid biosynthesis is very promising, since it is absent in mammals and its malfunction in fungi leads to attenuated virulence in an animal model. Knowledge about the metabolic steps that can be disrupted and how to block metabolic processes, such as sulfur uptake and sulfur amino acid biosynthesis may be a way to find therapeutic solutions for invasive fungal infections.

## Material and methods

### Strains, plasmids, reagents, and media

The plasmids, strains, and primers used in this work are listed in Supplementary Table S1, S2, and S3, respectively. Routine growth was carried out on YEPD (1% yeast extract, 2% bacto-peptone, 2% glucose). Synthetic dextrose (SD) was prepared with yeast nitrogen base, YNB (0.67 g/L yeast nitrogen base with or w/o amino acid and ammonium sulfate, depending on experimental design, 2% glucose). Growth was carried out at 30 °C, unless specified otherwise. Growth on liquid medium was conducted with 150 rpm on a rotary shaker. Sulfur amino acids were supplemented at 20 mM concentration each separately or in combination according to experimental design.

### Strain construction

#### *GFP*-*MET*3 (pRCP120)

Amplification of the *C. neoformans MET*3 gene was performed by PCR using H99 gDNA as the template from the start codon (ATG) to the stop codon (TGA), including approximately 300 bp of the gene terminator. This construction design allowed the Gfp fluorescent protein coding sequence present in the cloning vectors to be transcribed in the same reading frame as the *MET*3 coding sequence, producing a fusion protein (Gfp-Met3). Forward (PRCP488) and reverse (PRCP489) primers (Supplementary Table S3) were used to amplify a 2442 bp fragment. After purification, the DNA fragment was cloned into pCN50 vector digested with *Bam*HI and *Spe*I, which confers resistance to the antibiotic G418 for selection in *C. neoformans*. After the ligation was transformed into *E. coli* cells (DH5α), recombinant clones were selected in LB supplemented with ampicillin. Resistant colonies were subjected to diagnostic PCR reactions to confirm the cloning of the insert into the vector. Plasmid DNA of the positive transformants was extracted with the QIAprep Spin Miniprep Kit plasmid DNA extraction kit (Qiagen, Germantown, MD, USA) according to the manufacture’s protocol and submitted to DNA sequencing to confirm the junction. One of these plasmids, named pRCP120, was digested with *Hind*III, yielding a linear fragment that was transformed into H99 strain by electroporation. Stable transformants resistant to G418, named CNU184 and CNU185, were submitted to further analysis by western blot and fluorescent microscopy.

#### *GFP*-*CYS*3 *met*3Δ::*Nat*^R^ strain construction

TRACE (Transient CRISPR-Cas9 Coupled with Electroporation) was used to generate a *met*3Δ::*Nat*^R^ in the host strain CNU080, which contains a *GFP-CYS*3/*Neo*^R^ allele integrated at its genome and expresses a Gfp-Cys3 fusion protein^6^. TRACE was performed according to previously published protocol^37,38^. In brief, Cas9 endonuclease gene under the control of GPD1 promoter was amplified by PCR from pYF24 as the template, kindly donated by Dr. Xiaorong Lin Laboratory. A 9 Kb band was purified by QiaQuick (Qiagen). The gRNA target site was designed in EuPaGDT (Eukaryotic Pathogen CRISPR guide RNA/DNA Design Tool) embedded in FungiDB site (http://grna.ctegd.uga.edu/). The target site was 20 nucleotides long, and the NGG sequence was excluded to avoid self-cleavage of the gRNA. The nucleotide sequence of the target site was fused at its 3’ end to the sequences of the guide RNA scaffold to create the forward primer. A reverse primer for gRNA was also created, and the primer pair was used to amplify part of guide RNA using pDD162 (AddGene) plasmid as the template^39^. The complementary sequence of the target site was incorporated in the reverse primer containing at its 3’ end complementary to sequences of the U6 promoter, and the forward primer to amplify the U6 promoter was also designed. The primer pair was used to PCR amplify the U6 promoter using *C. neoformans* strain JEC21 genomic DNA. The two PCR amplified fragments were used as the template in a second PCR reaction and joined together using nested primers, generating the 20 nucleotides target site fused to the guide DNA scaffold driven by the U6 promoter. The 300 bp fusion product was purified with QiaQuick. The *met*3Δ::*Nat*^R^ deletion construct containing 500 bp of the promoter and terminator regions of the *MET*3 gene flanking the Noursethricin resistance gene driven by actin promoter was made by overlapping PCR as previously described^40^. All three DNA fragments encoding the gRNA, Cas9, and deletion construct were purified, eluted in water, and introduced into strain CNU080 (*GFP-CYS*3, *Neo*^R^) by electroporation according to the protocol previously published^38^. Transformants resistant to nourseothricin were transferred to synthetic dextrose medium with and without 10 mM of sulfur amino acids. Transformants were also submitted to diagnostic PCR to identify homologous recombination at the *MET*3 locus. Two of the auxotroph mutants that were positive in the diagnostic PCR were designated CNU153 and CNU183, tested for methionine and cysteine requirements and shown to be complemented by 10 mM of both amino acids separately and in combination. Furthermore, CNU153 and CNU183 continued to be G418 resistant after *MET*3 gene deletion, which guaranteed the retention of the resistance marker associated to *GFP-CYS*3 allele.

### Bioinformatics

Sequences relative to *MET*3 gene (CNAG_04215) were recovered from FungiDB database (https://fungidb.org/fungidb/app/). The amino acid sequence was used as a query to search for homologues in other organisms by BLASTp algorithm (https://blast.ncbi.nlm.nih.gov/Blast.cgi). Protein motifs and domains were identified by MotifScan database (https://myhits.sib.swiss/cgi-bin/motif_scan), which contains Prosite and Pfam databases. Amino acid sequence alignments were done by Lasergene MegaAlign software (DNA Star) by ClustalW algorithm. Schematic representation of protein was done by DOG 1.0: Illustrator of Protein Domain Structures software^41^.

### Two-hybrid experiment

#### *DB::MET3* (pRCP106)

The pGBKT7 vector was digested with *Bam*HI and *Eco*RI endonucleases according to the manufacturer’s instructions (ThermoFisher). The linear plasmid was purified with QiaQuick (Quiagen). *MET*3 cDNA (1746 bp) was amplified by RT-PCR with specific primers (Supplementary Table S3) with SuperScript II reverse transcriptase kit (ThermoFisher) from total RNA extracted from H99 as described before^42^. Bands of the correct size were excised from agarose gel, purified with QIAquick Gel extraction kit (Qiagen), cloned into linear pGBKT7 with In-fusion kit (Takara Clontech), and introduced in *E. coli* DH5α strains. Colonies resistant to kanamycin were harvested and submitted to colony PCR with the appropriated primer pair (Supplementary Table S3) to allow identification of the recombinant ones that carry the *MET*3 cDNA. The clones containing the correct insert were found to be identical, were identified by codes (pRCP106, pRCP107, and pRCP108), and used in subsequent experiments. All prey plasmids (pGADT7) used in the two-hybrid experiments were described in our previous work^6^ and are listed in Supplementary Table S1.

#### *DB::MET3ΔLZ* (pRCP128 and pRCP132)

The *MET*3Δ*lz* allele was obtained by deleting 66 nucleotides of the sequence (position 100 to 165) encoding the leucine zipper (22 amino acids, ^34^I to L^55^). In brief, two PCR fragments flanking the sequence encoding the leucine zipper were amplified (99 and 1581 bp) with specific primer pairs (Supplementary Table S3 and supplementary Fig. S2). The two DNA fragments were joined by overlapping PCR generating a single DNA fragment of 1680 bp without the coding sequence for the atypical leucine zipper. This DNA was cloned into pGBKT7 digested with *Bam*HI and *Eco*RI endonucleases according to the manufacturer’s instructions (ThermoFisher). Gibson Assembly® cloning kit (New England Biolabs Inc.) was used to assemble the vector and the insert into a single molecule, following the manufacturer’s instructions. An aliquot of the assembly reaction was transformed into *E. coli* cells (Stellar competent cells, Clontech Takara). Colonies resistant to kanamycin were confirmed by diagnostic PCR, restriction enzyme digestion, and Sanger sequencing (Supplementary Fig. S3). Two clones named pRCP128 and pRCP132 were used in *S. cerevisiae* two-hybrid assays.

#### Two-hybrid assay

Plasmids (bait and prey) were introduced in *S. cerevisiae* Y2HGold, which contains the 4 reporter genes (*Ade*2, *His*3, *Mel*1, and *Aur*1-C). The transformation was made by lithium acetate and PEG protocol according to MatchMaker manual (Clontech). In yeast, pGBKT7 (Trp) and pGADT7 (Leu) are selected by auxotroph complementation of Y2HGold in synthetic dextrose medium minus tryptophan or leucine (SDO, Single Drop Out). In the case of co-transformation, the selection was in synthetic dextrose minus tryptophan and leucine (Double Drop Out, DDO). Synthetic dextrose lacking tryptophan and leucine but added with X-α-Gal and Aurobasidin (DDO/X/A) is suitable to test 2 reporter genes (*Aur*1-C and *Mel*1, respectively). QDO is SD lacking tryptophan, leucine, adenine, and histidine, testing 2 reporters (*Ade*2 and *His*3), and QDO/X/A is the most stringent medium to test for protein interactions, lacking tryptophan, leucine, adenine, and histidine and with aurobasidin and X-α-Gal added. All plasmids were tested for auto activation of the system, which were carried out in SDO/X/A (synthetic dextrose minus tryptophan or leucine added with aurobasidin and X-α-Gal). In addition, toxicity was evaluated in SDO (synthetic dextrose minus tryptophan or leucine).

Protein interaction tests were conducted according to MatchMaker Yeast Two-Hybrid System protocol (Clontech). In brief, combinations of bait and prey plasmids were introduced in Y2HGold and plated on DDO, where all transformations should yield colonies. Transformants that grew on DDO were transferred to reporter test medium: QDO, QDO/X, and QDO/X/A. In all experiments, the positive and negative controls provided by MatchMaker Yeast Two-Hybrid System (Clontech) were used.

### Western blot

Protein extract was obtained and separated on SDS-PAGE as described before^6^. The gels were equilibrated in transfer buffer (48 mM Tris, 39 mM glycine, 20% methanol) and proteins were transferred to nitrocellulose membranes on Trans-Blot® SD Semi-Dry Electrophoretic Transfer Cell (BioRad) 15 V for 1 h. The membrane was blocked with 5% non-fat dry milk in TBS (10 mM Tris, 150 mM NaCl, pH 7.4) for 1 h at room temperature. The primary antibody (mouse anti-GFP ThermoFisher, 1:7000 dilution) was incubated overnight at 4 °C in 1% BSA. After three of 5-min washes each in TBST (TBS with 0.1% Tween 20), the secondary antibody (goat anti-mouse-HRP, Cell Signaling Technology 1:2000 dilution) was incubated in TBST with 5% non-fat dry milk for 1 h at room temperature followed by three 5-min washes each in TBST. Detection of chemiluminescent bands was performed by SuperSignal West Pico PLUS Substrate (ThermoFisher) using ImageQuant LAS 4000 system (GE). Loading control was done with rabbit anti-Histone H3 antibody (1:2000) and anti-rabbit secondary antibody HRP-linked (1:2000). Protein extracts and western blots were made in triplicates.

### Fluorescent microscopy

Strains containing Gfp-Met3 or Gfp-Cys3 alleles were induced in several nutritional conditions depending on experimental design. Cells were grown overnight in 5 mL YEPD at 30 °C with 150 rpm rotation, washed with PBS three times, then diluted to OD_600_ = 0.6 (5 mL), and incubated in various nutritional conditions at 30 °C for 2 h. A 1 mL aliquot was removed for microscopy analysis. The cells were fixed in 4% formaldehyde (Sigma) (v/v) diluted in 100 mM potassium phosphate and 0.5 mM MgCl_2_ for 10 min at 30 °C and washed twice with 1X PBS. Glass slides were prepared with 4 µL of ProLong with NuckBlue antifade (Thermo Scientific) and 6 µL of the processed sample. Cells were viewed by direct fluorescent microscopy using an Olympus BX51M microscope and analysis was performed using Olympus CellSens, PhotoShop CS6, and ImageJ software. Percentages of nuclear Gfp-Cys3 were obtained by counting the cells in which Gfp and DAPI were overlaid (n > 100 cells/replicate). All microscopy experiments were done in triplicates.

### qPCR

Total RNAs were obtained from strains grown overnight in liquid medium under 150 rpm agitation at 30 °C. RNA extraction was performed as described before^43^. RevertAid H minus First Strand cDNA synthesis kit (Thermo Scientific) was used to obtain cDNA with Oligo dT and random hexamer primers from 5 μg of total RNA. Diluted cDNA templates (1:10) were amplified with 800 ηM target primers, 300 ηM GPDH1 (Glyceraldehyde-3-phosphate dehydrogenase) internal control primers, and 1X Syber Green (Evagreen®). Quantification of the transcript levels was performed in StepOne thermo cycler (Applied Biosystems) using the 2^ΔΔCT^ method normalizing against GPDH1, as previously described^44^. An analysis of variance was performed by Tukey’s multiple comparison test using GraphPad Prism 7.0 software, and *p* values lower than 0.05 were considered statistically significant.

## Supplementary Information

Supplementary Information.

## Data Availability

All the relevant data in the paper and its Supplementary Information files.
