# Supplementary material for: ATP sulfurylase atypical leucine zipper interacts with Cys3 and calcineurin A in the regulation of sulfur amino acid biosynthesis in Cryptococcus neoformans
Source: Sci Rep. 2023 Jul 20;13:11694. doi: 10.1038/s41598-023-37556-5 (PMC10359356; doi:10.1038/s41598-023-37556-5)
Supplement: Supplementary file 1 — Supplementary Information. [file 41598_2023_37556_MOESM1_ESM.docx]

**ATP sulfurylase atypical leucine zipper interacts with Cys3 and calcineurin A in the regulation of sulfur amino acid biosynthesis in *Cryptococcus neoformans***

**Supplementary Information**

Authors:

Jeyson Pereira da Silva^1^; Mariana Reis Meneghini^1^; Ronaldo Silva Santos^1^; Verônica Lira Alves^1^; Kevin Felipe da Cruz Martho^1^; Marcelo Afonso Vallim^1^; Renata Castiglioni Pascon^1+^

^1^ Affiliation:

Universidade Federal de São Paulo, Campus Diadema, Rua São Nicolau, 210

Diadema, SP - Brazil

ZIP code: 09913-030

^+^ Corresponding author: [renata.pascon@unifesp.br](mailto:renata.pascon@unifesp.br)

Pathwords:

ATP sulfurylase, *Cryptococcus neoformans*, sulfur amino acid biosynthesis, protein interaction, leucine zipper

Supplementary figure S1: Figure 1: *C. neoformans* Met30 protein domains and its interaction with Cys3 by *S. cerevisiae* two-hybrid assay. (A) The silver bars represent the Met30 protein of *C. neoformans* and *S. cerevisiae* with F-box domain (in pink) at the N-terminus and WD40 motifs (in blue) found at the C-terminus. Image was generated by DOG 1.0: Illustrator of Protein Domain Structures software 42; (B) Images show representative colonies of *S. cerevisiae* Y2HGold strain expressing pairs of bait and prey fusion proteins; + and - are positive and negative controls transformed with pGBKT7-53 (bait) and pGADT7-T (prey) and pGBKT-Lam (bait) and pGADT7-T (prey), respectively. Plasmids were provided by Match Maker kit (Clontech). Three independent clones of Y2HGold strain contain plasmids pRCP109 (DB::MET30) and pRCP99 (AD::CYS3), pRCP109 (DB::MET30) and pRCP100 (AD::CYS3), pRCP111 (DB::MET30) and pRCP099 (AD::CYS3), respectively. Strains were inoculated on DDO medium = double drop out (no reporter gene tested), QDO medium = quadruple drop out (Ade2 and His3 reporter genes tested), QDO/X medium = quadruple drop out plus X-α-Gal (Ade2, His3, and Mel1 reporter genes tested), QDO/X/A medium = quadruple drop out plus X-α-Gal and aureobasidin (Ade2, His3, Mel1, and AuriC reporter genes tested). Plates were incubated for 96 hours at 30 °C.

Supplementary figure S2: ATP sulfurylase domains. The catalytic domain, known for its α-β phosphodiesterase activity is followed by an ATP binding site (VxAFQxRNPxHxG/AH). In C. neoformans these domains are between V200 and H213. Also, the alignment allowed the identification of a PP-loop between amino acids H283-G303, which is used for binding and presenting of ATP as substrate for sulfurylation (Supplementary Figure 1). Localization of the putative LxVP calcineurin binding sites. Its conservation and location may vary considerably among calcineurin targets. C. neoformans ATP sulfurylase has 4 putative LxVP targets, in three of them the consensus is DxVP and one of them is ExVP. All four motifs are downstream of the putative PxIxIT motif (85FPIPIT90).


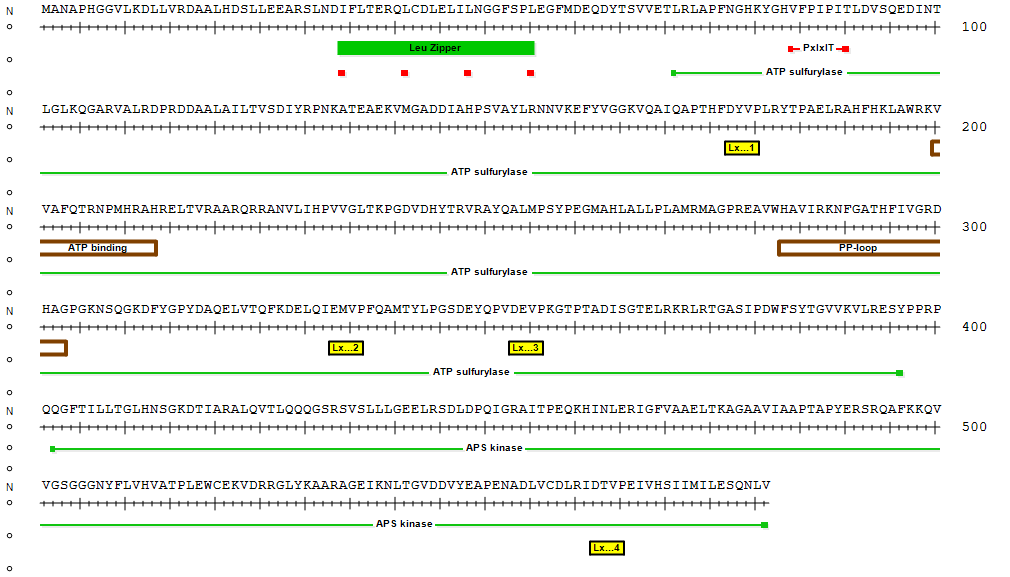


Supplementary figure S3: Sequence alignment showing leucine zipper deletion in pRCP128 and pRCP132 against wild-type allele.

Supplementary figure S4: Ratio of Gfp-Cys3 intensity in the nucleus/cytoplasm in three different nutritional condition considering 3 strains: CNU080 is the wild type and CNU153 and CNU183 are the met3Δ mutants. The differences observed are not statistically significant.

Supplementary figure S5: Original membrane image presented in the western blot (Figure 5A). The first 3 lanes were cropped.

Supplementary figure S6: Original western blot membrane that originated Figure 5A, detecting Gfp-Met3. Anti-Gfp antibody.





Supplementary figure S7: Original western blot membrane that originated Figure 5A - normalizer Histone H3. Anti-Histone antibody.





Supplementary figure S8: Original western blot membrane that originated Figure 6B, detecting Gfp-Cys3. Anti-Gfp antibody.





Supplementary figure S9: Original western blot membrane that originated Figure 6B, detecting the normalizer protein Histone H3. Anti-histone antibody.





Supplementary figure S10: Original western blot membrane that originated Figure 7A, detecting Gfp-Met3 in cna1Δ strain. Anti-Gfp antibody.





Supplementary figure S11: Original western blot membrane that originated Figure 7A, detecting the normalize Histone H3 in cna1Δ strain. Anti-histone antibody.





Supplementary table S1: Plasmids used in this work.

| **Plasmid code** | **Vector** | **Gene** | **E. coli antibiotic resistance** | **Fungal Gene marker** | **Source** |
| --- | --- | --- | --- | --- | --- |
| pRCP088 | pGADT7 | *cDNA GPP2* | Ampicillin | LEU2 | De Melo et al., 2019. |
| pRCP094 | pGADT7 | *cDNA CNA1ΔC* | Ampicillin | LEU2 | De Melo et al., 2019. |
| pRCP096 | pGADT7 | *cDNA CNB1* | Ampicillin | LEU2 | De Melo et al., 2019. |
| pRCP099 | pGADT7 | *cDNA CYS3* | Ampicillin | LEU2 | De Melo et al., 2019. |
| pRCP100 | pGADT7 | *cDNA CYS3* | Ampicillin | LEU2 | De Melo et al., 2019. |
| pRCP106 | pGBKT7 | *cDNA MET3* | Kanamycin | TRP1 | This work |
| pRCP109 | pGBKT7 | *cDNA MET30* | Kanamycin | TRP1 | This work |
| pRCP110 | pGBKT7 | *cDNA MET30* | Kanamycin | TRP1 | This work |
| pRCP120 | pCN50 | *GFP::MET3* | Amp/Kan | G418 | This work |
| pRCP128 | pGBKT7 | *cDNA MET3ΔZP* | Kanamycin | TRP1 | This work |
| pRCP132 | pGBKT7 | *cDNA MET3ΔZP* | Kanamycin | TRP1 | This work |

Supplementary table S2: Strains used in this work.

| **Strain Code** | **Background strain** | **Gene marker** | **Origen** |
| --- | --- | --- | --- |
| *C. neoformans* | | | |
| CNU080 | H99 | *pHis:GFP::CYS3 NEO^R^* | De Melo et al., 2019 |
| CNU153 | CNU080 | *met3Δ::NAT^R^* | This work |
| CNU183 | CNU080 | met3Δ::NAT^R^ | This work |
| CNU184 | H99 | GFP-MET3 (pRCP120) | This work |
| CNU185 | H99 | GFP-MET3 (pRCP120) | This work |
| CNU186 | CNU166 | cna1Δ::HPH^R^, GFP::MET3::NEO^R^ (pRCP120) | This work |
| CNU188 | CNU166 | cna1Δ::HPH^R^, GFP::MET3::NEO^R^ (pRCP120) | This work |
| *S. cerevisiae* | | | |
| YL018 | Y2HGOLD | pGBKT7:Met3 (pRCP106) and pGADT7:Cys3 (pRCP099) | De Melo et al., 2019 |
| YL019 | Y2HGOLD | pGBKT7:Met3 (pRCP106) and pGADT7:Cna1-ΔC (pRCP094) | De Melo et al., 2019 |
| YL026 | Y2HGOLD | pGBKT7:Met3ΔLZ (pRCP128) and pGADT7:Cna1-ΔC (pRCP094) | This work |
| YL027 | Y2HGOLD | pGBKT7:Met3ΔLZ (pRCP132) and pGADT7:Cna1-ΔC (pRCP094) | This work |
| YL028 | Y2HGOLD | pGBKT7:Met3ΔLZ (pRCP128) and pGADT7:Cys3 (pRCP099) | This work |
| YL029 | Y2HGOLD | pGBKT7:Met3ΔLZ (pRCP132) and pGADT7:Cys3 (pRCP099) | This work |
| Positive control | Y2HGOLD | pGBKT7-53 and pGADT7-T | Clonteck |
| Negative control | Y2HGOLD | pGBKT7-Lam and pGADT7-T | Clonteck |

Supplementary table S3: Primers used in this work.

| **Primer Code** | **Sequence** | **F or R** | **Use** |
| --- | --- | --- | --- |
| PRCP426 | CATTCGTTCAGCAACCATTGC | F | MET3 deletion (CNAG_04215) |
| PRCP427 | CTCCAGCTCACATCCTCGCATTTTGCTGTTGTAAATGAGGTTTG | R | MET3 deletion (CNAG_04215) |
| PRCP428 | CAAACCTCATTTACAACAGCAAAATGCGAGGATGTGAGCTGGAG | F | MET3 deletion (CNAG_04215) |
| PRCP429 | ACGCTACAAGCTCTTTTTTTGATGAAGAGATGTAGAAACTAGCTTCC | R | MET3 deletion (CNAG_04215) |
| PRCP430 | GGAAGCTAGTTTCTACATCTCTTCATCAAAAAAAGAGCTTGTAGCGT | F | MET3 deletion (CNAG_04215) |
| PRCP431 | GCCTGGTACCGCATTGACC | R | MET3 deletion (CNAG_04215) |
| PRCP432 | GTTATCATCGTGCAACTG | F | MET3 deletion confirmation (CNAG_04215) |
| PRCP435 | CCATGGAGGCCGAATTCATGGCCAACGCTCCTCACG | F | MET3 cloning in pGBKT7 |
| PRCP436 | GCAGGTCGACGGATCCTTAAACAAGATTCTGGCTCTC | R | MET3 cloning in pGBKT7 |
| PRCP443 | CCATGGAGGCCGAATTCATGTCTCCTTCTGCGCCACC | F | MET30 cloning in pGBKT7 |
| PRCP444 | GCAGGTCGACGGATCCTTATACAATGGCAGGGGTCG | R | MET30 cloning in pGBKT7 |
| PRCP488 | GAGCTGTACGAGCTCGGATCCATGGCCAACGCTCCTCACG | F | GFP-MET3 cloning in pCN50 |
| PRCP489 | GGCGGCCGTTACTAGTGGATCCGCGTGGGTTCGAATCCCAC | R | GFP-MET3 cloning in pCN50 |
| PRCP495 | GGTGACGCTGTGAGAGTGG | F | Cas9 amplification from pYF24 |
| PRCP496 | GGGCCCCTCTTCACGTGG | R | Cas9 amplification from pYF24 |
| PRCP497 | GCAAGGGCAGCATCGTCACGAACAGTATACCCTGCCGGTG | R | U6 promoter reverse with MET3 target site |
| PRCP498 | CGTGACGATGCTGCCCTTGCGTTTTAGAGCTAGAAATAGCAAGTT | F | gRNA forward with MET3 target site |
| PRCP504 | GCTCGTCCATGAAACCTTCGTCATTCAAGCTACGAGCC | R | MET3 leucine zipper deletion |
| PRCP505 | GGCTCGTAGCTTGAATGACGAAGGTTTCATGGACGAGC | F | MET3 leucine zipper deletion |
| PRCP530 | CTACACTTCCGTCGTTGAGAC | F | qPCR MET3 |
| PRCP531 | TGTCAGAGACAGTAAGGATAGC | R | qPCR MET3 |
| PRCP424 | GATCTTGTGGCTGGTATCACC | F | qPCR SUL1 |
| PRCP425 | GGTTGAGGGATGAACTCAACG | R | qPCR SUL1 |
| MAV240 | AGTATGACTCCACACATGGTCG | R | qPCR GPDH |
| MAV241 | AGACAAACATCGGAGCATCAGC | F | qPCR GPDH |
